# Supplementary material for: Brain Invasion along Perivascular Spaces by Glioma Cells: Relationship with Blood–Brain Barrier
Source: Cancers (Basel). 2019 Dec 19;12(1):18. doi: 10.3390/cancers12010018 (PMC7017006; doi:10.3390/cancers12010018)
Supplement: Supplementary file 1 [file cancers-12-00018-s001.zip › Appendix.docx]

**SUPPLEMENTARY MATERIALS AND METHODS**

***Intracranial xenografts of GFP expressing U87MG and GSC1 cells***

Immunosuppressed athymic rats (male, 250-280 g; Charles River, Milan, Italy) were anesthetized with intraperitoneal injection of diazepam (2 mg/100 g) followed by intramuscular injection of ketamine (4 mg/100 g). Animal skulls were immobilized in a stereotactic head frame and a burr hole was made 3 mm right of the midline and 2 mm anterior to the bregma. The tip of a 10 μl-Hamilton microsyringe was placed at a depth of 5 mm from the dura and 2×10^4^ of either GFP+ U87MG cells or GFP+ GSC1 were slowly injected. After grafting, the animals were kept under pathogen-free conditions and observed daily for neurological signs. After survivals ranging from 14 days to 16 weeks, the rats were deeply anesthetized and transcardially perfused with 0.1 M PBS (pH 7.4), then treated with 4% paraformaldehyde in 0.1 M PBS. The brain was removed and stored in 30% sucrose buffer at 4° C for three days.

***Antigen retrieval and auto-fluorescence removal in brain tumor xenografts and human specimens***

The antigen masking effect of formalin fixation of human brain tissues required specific antigen retrieval procedures before immunostaining. To unmask CRMP5, ZO-1, and Claudin5 antigens, a Citrate Buffer antigen retrieval Solution (CBS; 10 mM citric acid, 0.05% Tween 20, pH 6.0) was used as follows. Human tissue sections were immersed for 30’ in CBS, pre-heated at 95-98° C, and then transferred at room temperature allowing CBS to cool. Sections were washed in PB and incubated with primary antibodies. To unmask IDH1 (R132H) antigen, sections were pre-treated with 0.3M glycine in PB pH 7.4 for 70’ at room temperature, extensively washed in PB, before incubation with the primary anti-IDH1 (R132H) antibody. Since antigen retrieval with CBS is performed at high temperature, it denatures GFP and suppresses its fluorescence [33,34], making it impossible to visualize the presence of GFP-expressing tumor cells. For this reason, rat slices treated with CBS were subsequently incubated with the primary mouse monoclonal anti-GFP antibody (1:100; Roche, Basel, Switzerland). The presence of lipofuscin granules in the central nervous system can mystify observations obtained by fluorescence microscopy because of its broad excitation and emission spectra, which overlaps with those of many fluorophores. To reduce human tissue auto-fluorescence, at the end of immunofluorescence procedures, sections were treated with 0.3% Sudan Black B (Sigma Aldrich) in ethanol 70% for 25-30 seconds at room temperature, followed by other 3 consecutive washing steps in PB (10’ each).

***Fluorescence microscopy and immunofluorescence of brain tumor xenografts***

The brains were serially sectioned (40 μm thickness) by a cryostat on the coronal plane. Sections were blocked in PB with 10 % BSA, 0.3 % Triton X-100 for 45 minutes and incubated overnight at 4 °C with primary antibodies in PB with 0.3 % Triton X-100 and 0.1% normal donkey serum (NDS). Monoclonal antibodies used were as follows, mouse anti-Glucose Transporter GLUT1 antibody (1:100; Abcam, Cambridge, UK), mouse anti-Rat Blood-Brain Barrier (Clone SMI-71; 1:500; Biolegend, San Diego, CA), mouse anti-Claudin-5 (1:100; Thermo Fisher Scientific, Waltham, MA). Polyclonal antibodies used were as follows, rabbit anti-Glucose Transporter GLUT1 antibody (1:200; NovusBio, Centennial, CO), rabbit anti-ZO-1 (1:100; Thermo Fisher Scientific, Waltham, MA), goat anti-GFAP (1:1000; Thermo Fisher Scientific, Waltham, MA), rabbit anti-GFAP (1:1000; Dako Italia, Milan, Italy). For detecting brain microvessels, sections were incubated overnight at 4°C in PB with 0.3 % Triton X-100 and 0.1% NDS with Lectin from *Lycopersicon esculentum* (tomato) biotin conjugate (1:500; Sigma-Aldrich, St. Louis, MO) together with primary antibodies. Slices were rinsed and incubated in PB containing 0.3% Triton X-100 with secondary antibodies for 2 hours at RT. Secondary antibodies used were as follows, Alexa Fluor 647 or 555 or 488 donkey anti-mouse, Alexa Fluor 488 or 555 or 647, donkey anti-rabbit secondary antibodies (1:500; Thermo Fisher Scientific, Waltham, MA), Alexa Fluor 488 or 555 donkey anti-goat antibodies (1:400; Thermo Fisher Scientific, Waltham, MA), Cy3 donkey anti-Rat (1:200, EMD Millipore, Billerica, MA, USA). For lectin immunostaining, sections were incubated for 2 hours at RT in PB containing 0.3 % Triton X-100 with streptavidin protein Alexa Fluor® 647 or 555 conjugate (1:200; Thermo Fisher Scientific, Waltham, MA). Before mounting, slices were incubated with 4,6-diamidino-2-phenylindole (DAPI, 1:4000; Sigma-Aldrich) for 10 minutes. In order to detect vascular permeability in U87MG and GSC rat xenografts, sections were incubated overnight at 4°C in PB 0.3 % Triton X-100 with Alexa Fluor 555 donkey anti-rat IgG (1:100; Abcam, Cambridge, UK) together with other primary antibodies. Integrity of BBB and density of tumor cells were assessed by quantification of SMI71 and GFP signals, respectively. Binary images created from confocal acquisitions were processed using NIH ImageJ software (<https://imagej.nih.gov/ij/>). Immunofluorescence was observed with a laser confocal microscope (Leica SP5 or Olympus FV1200). Image analysis was performed with Leica Application Suite X software and ImageJ software (NIH).
