# Supplementary material for: Brain Invasion along Perivascular Spaces by Glioma Cells: Relationship with Blood–Brain Barrier
Source: Cancers (Basel). 2019 Dec 19;12(1):18. doi: 10.3390/cancers12010018 (PMC7017006; doi:10.3390/cancers12010018)
Supplement: Supplementary file 1 [file cancers-12-00018-s001.zip › Supplementary Table S1.docx]

Supplementary Table S1. Summary of Findings of BBB Immunostaining on Brain Xenografts

| **Rat Brain** | **SMI-71*** | **Glut-1*** | **ZO-1*** | **IgG°** |
| --- | --- | --- | --- | --- |
| **Normal** | **+++** | **+++** | **+++** | **-** |
| **U87MG Xenograft**   - Tumor Bulk - Peritumor Brain | **-**  **-** | **+**  **++** | **-/+**  **+** | **++**  **++** |
| **GSC1 Xenograft**   - Tumor Bulk - Peritumor Brain | **+**  **++** | **++/+**  **+++/++** | **+**  **++** | **+**  **-** |

(*) +++, immunoreaction encicling the entire circumference of the vessel;

++, immunoreaction encicling at least 50% of the circumference of the vessel;

+, immunoreaction encicling less than 50% of the circumference of the vessel;

-, immunoreaction absent.

(°) +++, strong immunoreaction;

++, mild immunoreaction;

+, light immunoreaction;

-, immunoreaction absent.
