# Supplementary material for: Brain Invasion along Perivascular Spaces by Glioma Cells: Relationship with Blood–Brain Barrier
Source: Cancers (Basel). 2019 Dec 19;12(1):18. doi: 10.3390/cancers12010018 (PMC7017006; doi:10.3390/cancers12010018)
Supplement: Supplementary file 1 [file cancers-12-00018-s001.zip › Supplementary Table S2.docx]

Supplementary Table S2. *Clinical and Pathological Features of Patients’ Tumors*

| **Case #** | **Age/Sex** | **Tumor Location** | **Histology (WHO grade)** | **Ki-67(%)** | **Molecular Profile (*)** | **Comment** |
| --- | --- | --- | --- | --- | --- | --- |
| 1 | 40/M | R Frontal | Astrocytoma (II) | 1 | IDH mut |  |
| 2 | 38/F | R Insular | Astrocytoma (II) | <1 | IDH mut |  |
| 3 | 36/M | R Frontal | Oligodendroglioma (II) | 2 | IDH mut, 1p19p codel |  |
| 4 | 31/M | L Frontal | Anaplastic astrocytoma (III) | 5 | IDH mut |  |
| 5 | 48/M | L Temporal | Anaplastic astrocytoma (III) | 3 | IDH mut |  |
| 6 | 46/F | L Frontal | Oligodendroglioma (III) | 10 | IDH mut, 1p19p codel |  |
| 7 | 31/M | R Frontal | Oligodendroglioma (III) | 2 | IDH mut, 1p19p codel |  |
| 8 | 51/M | L Temporal | Glioblastoma (IV) | 15 | IDH wt, EGFRvIII + |  |
| 9 | 50/M | L Parietal | Glioblastoma (IV) | 3 | IDH wt, EGFRvIII + | Recurrent |
| 10 | 62/F | R Frontal | Glioblastoma (IV) | 25 | IDH wt, EGFRvIII + |  |
| 11 | 71/M | R Occipital | Glioblastoma (IV) | 25 | IDH wt, EGFRvIII - |  |
| 12 | 70/M | R Occipital | Glioblastoma (IV) | 5 | IDH wt, EGFRvIII - | Recurrent |
| 13 | 48/M | R Occipital | Glioblastoma (IV) | 30 | IDH wt, EGFRvIII + | Recurrent |
| 14 | 66/M | R Temporal | Glioblastoma (IV) | 20 | IDH wt, EGFRvIII - |  |
| 15 | 70/F | L Frontal | Glioblastoma (IV) | 20 | IDH wt, EGFRvIII + |  |
| 16 | 65/M | L Frontal | Glioblastoma (IV) | 15 | IDH mut, EGFRvIII - |  |
| 17 | 75/M | R Frontal | Glioblastoma (IV) | 20 | IDH wt, EGFRvIII - |  |
| 18 | 52/F | L Frontal | Glioblastoma (IV) | 25 | IDH wt, EGFRvIII + |  |
| 19 | 70/F | R Temporal | Glioblastoma (IV) | 15 | IDH wt, EGFRvIII + |  |
| 20 | 67/F | R Temporal | Glioblastoma (IV) | 20 | IDH wt, EGFRvIII + | Recurrent |
| 21 | 75/F | R Frontal | Glioblastoma (IV) | 20 | IDH wt, EGFRvIII - |  |

*, IDH mutation and EGFRvIII expression were assessed as described in D'Alessandris, Q.G., et al. The clinical value of patient-derived glioblastoma tumorspheres in predicting treatment response. *Neuro Oncol.* **2017,** *19,* 1097−1108.
