# Supplementary material for: Brain Invasion along Perivascular Spaces by Glioma Cells: Relationship with Blood–Brain Barrier
Source: Cancers (Basel). 2019 Dec 19;12(1):18. doi: 10.3390/cancers12010018 (PMC7017006; doi:10.3390/cancers12010018)
Supplement: Supplementary file 1 [file cancers-12-00018-s001.zip › Supplementary Table S3.docx]

Supplementary Table S3. *Summary of Findings of BBB Immunostaining in Gd-enhanced and Gd-unenhanced Regions of Human Glioma*

| **Human Brain** | **Glut-1*** | **ZO-1*** | **Claudin-5*** | **IgG°** |
| --- | --- | --- | --- | --- |
| **Normal** | **+++** | **+++** | **+++** | **-** |
| **Astrocytoma (II)**   - Gd-unenhanced | **+++** | **++** | **+++** | **-** |
| **Oligodendroglioma (II)**   - Gd-unenhanced | **+++/++** | **++** | **+++** | **-** |
| **Astrocytoma (III)**   - Gd-unenhanced - Gd-enhanced | **++**  **+** | **++**  **+** | **+++**  **++** | **-**  **++** |
| **Oligodendroglioma (II)**   - Gd-unenhanced - Gd-enhanced | **++**  **+** | **++**  **+** | **+++**  **++** | **-**  **++** |
| **Glioblastoma (IV)**   - Gd-unenhanced - Gd-enhanced | **++**  **+** | **++**  **-** | **+++/++**  **++/+** | **+**  **+++** |

(*) +++, immunoreaction encicling the entire circumference of the vessel;

++, immunoreaction encicling at least 50% of the circumference of the vessel;

+, immunoreaction encicling less than 50% of the circumference of the vessel;

-, absent immunoreaction.

(°) +++, strong immunoreaction;

++, mild immunoreaction;

+, light immunoreaction;

-, absent immunoreaction.
