# Supplementary figures and images for: Brain Invasion along Perivascular Spaces by Glioma Cells: Relationship with Blood–Brain Barrier
Source: Cancers (Basel). 2019 Dec 19;12(1):18. doi: 10.3390/cancers12010018 (PMC7017006; doi:10.3390/cancers12010018)

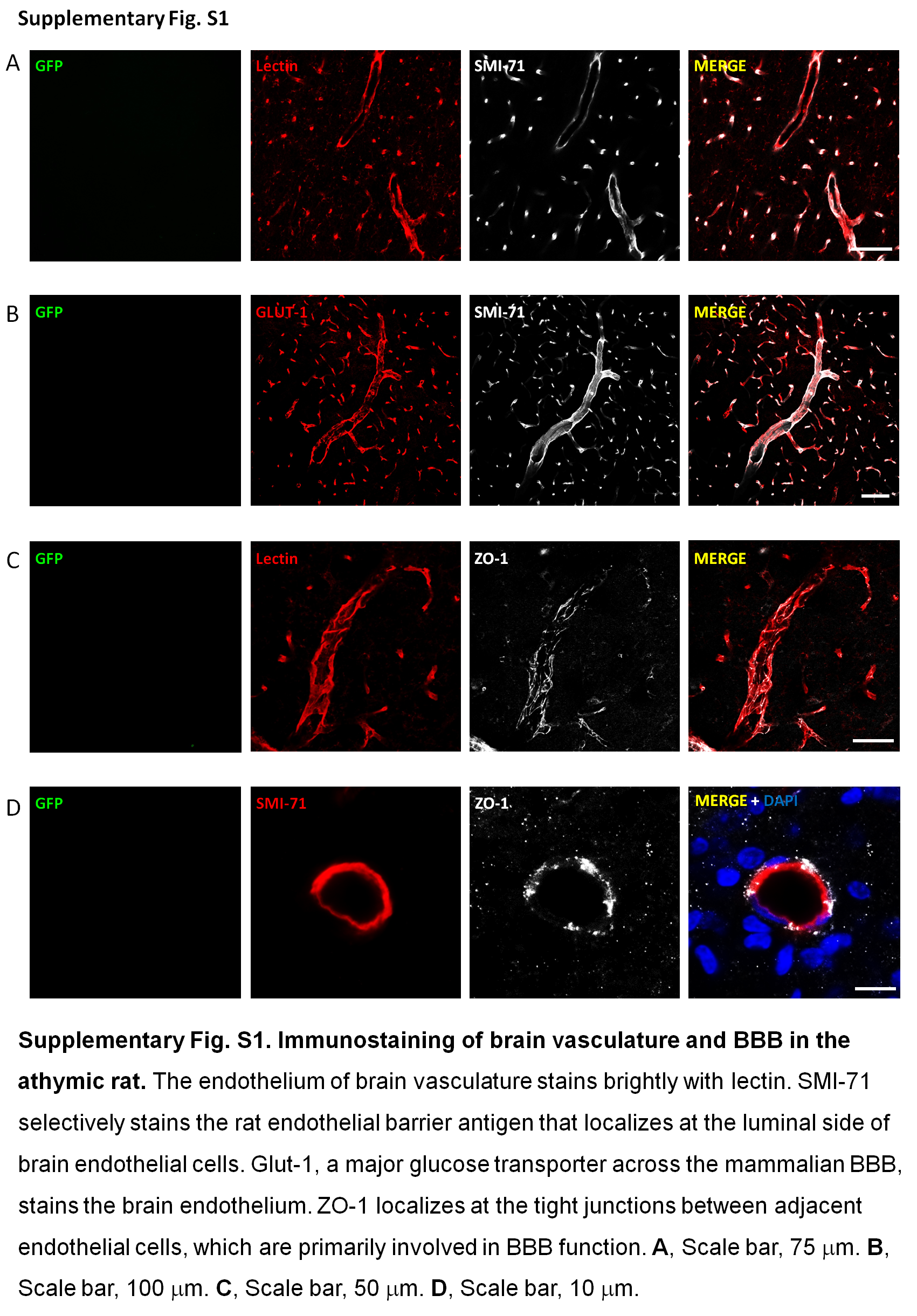

Supplement: Supplementary file 1 [file cancers-12-00018-s001.zip › Supplementary Figure S1.tif]

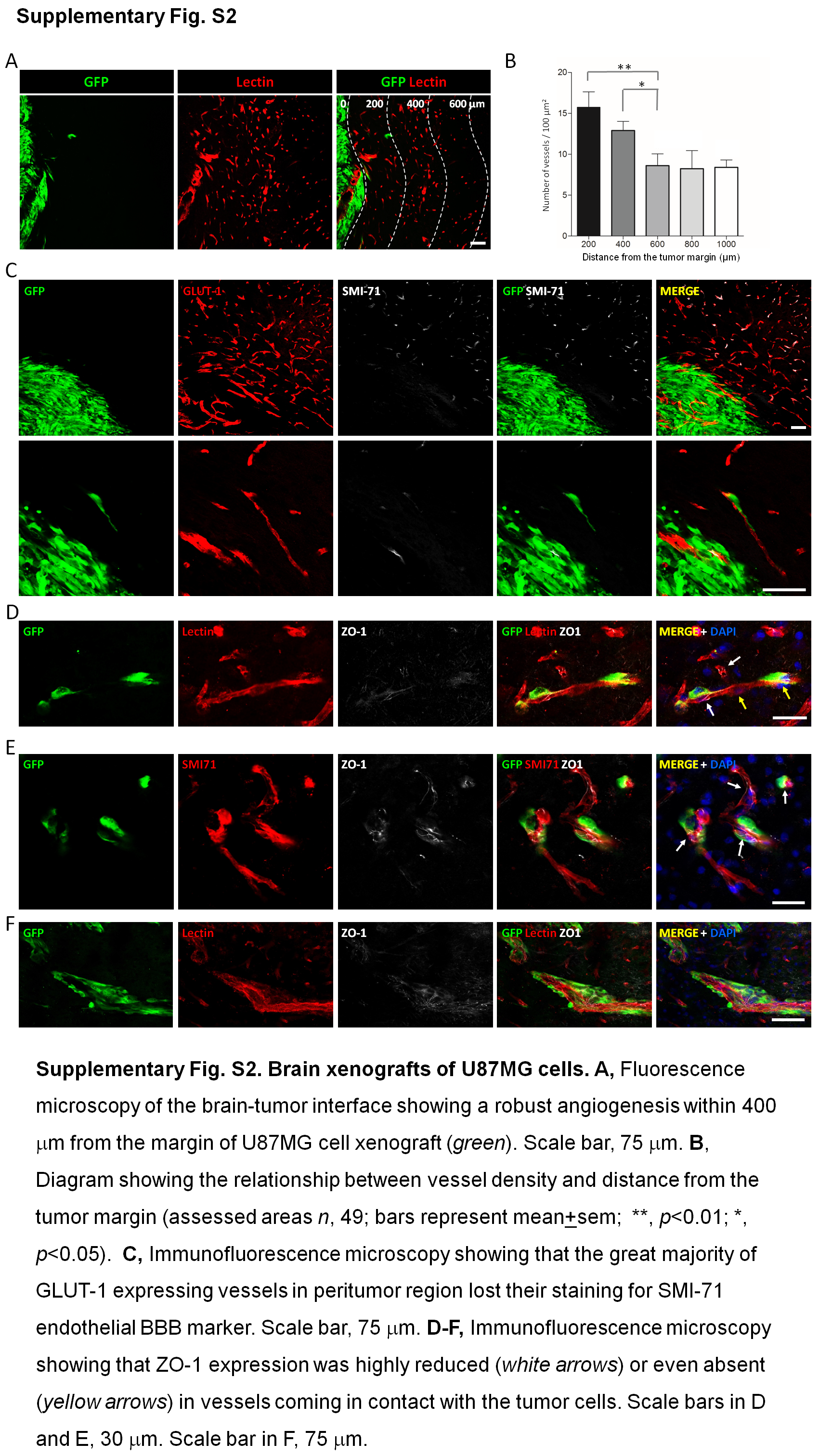

Supplement: Supplementary file 1 [file cancers-12-00018-s001.zip › Supplementary Figure S2.tif]

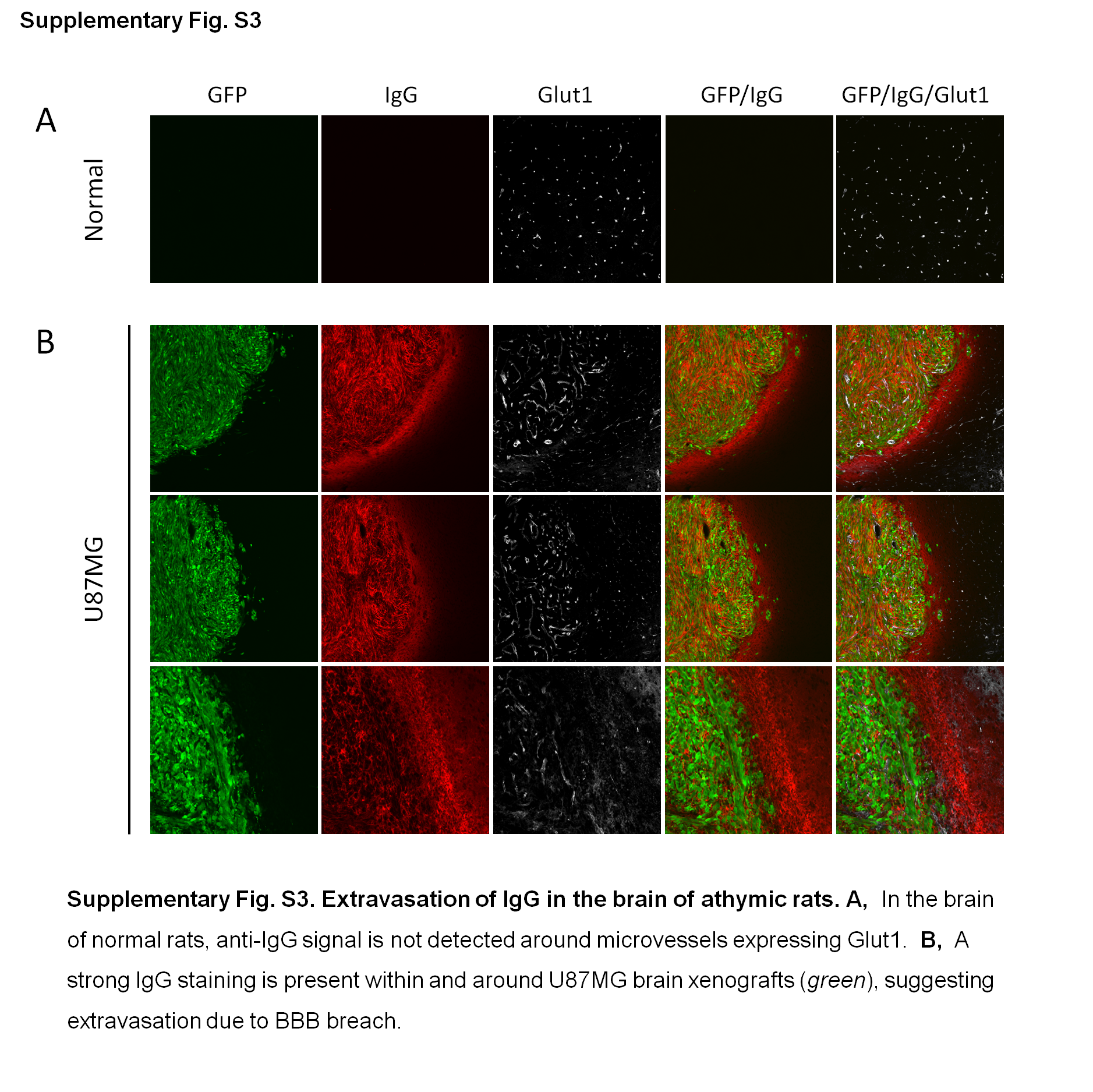

Supplement: Supplementary file 1 [file cancers-12-00018-s001.zip › Supplementary Figure S3.tif]

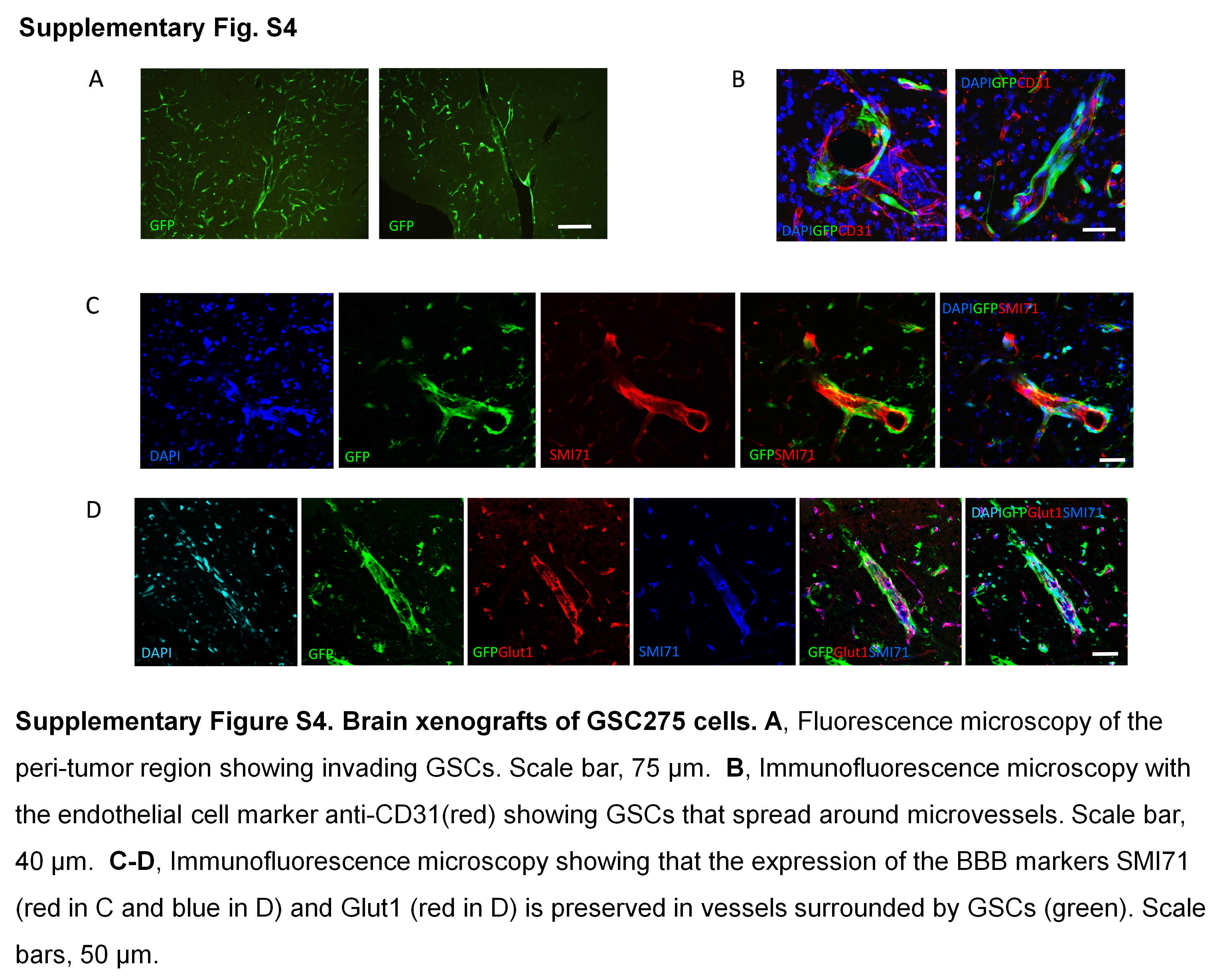

Supplement: Supplementary file 1 [file cancers-12-00018-s001.zip › Supplementary Figure S4.tif]

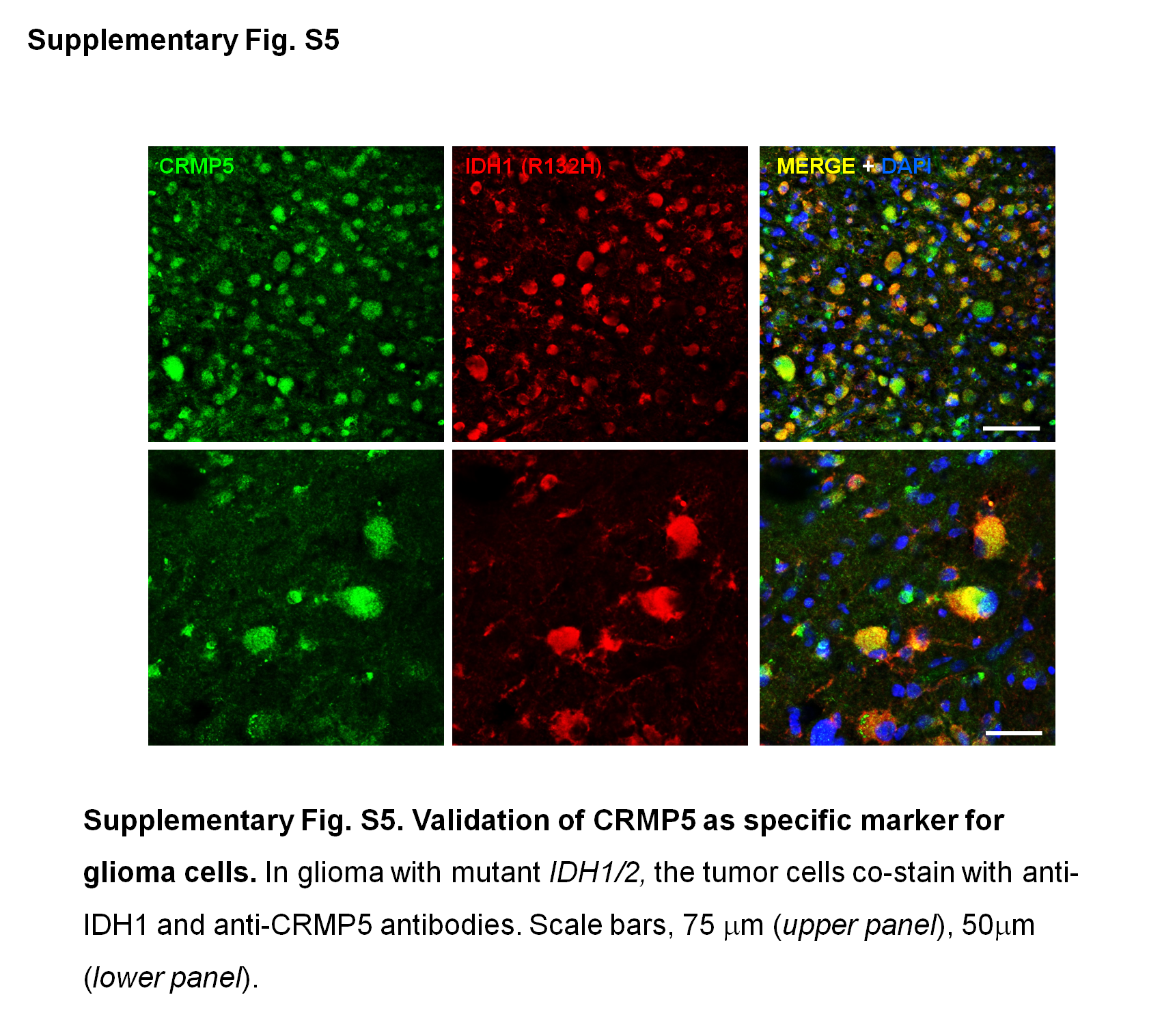

Supplement: Supplementary file 1 [file cancers-12-00018-s001.zip › Supplementary Figure S5.tif]

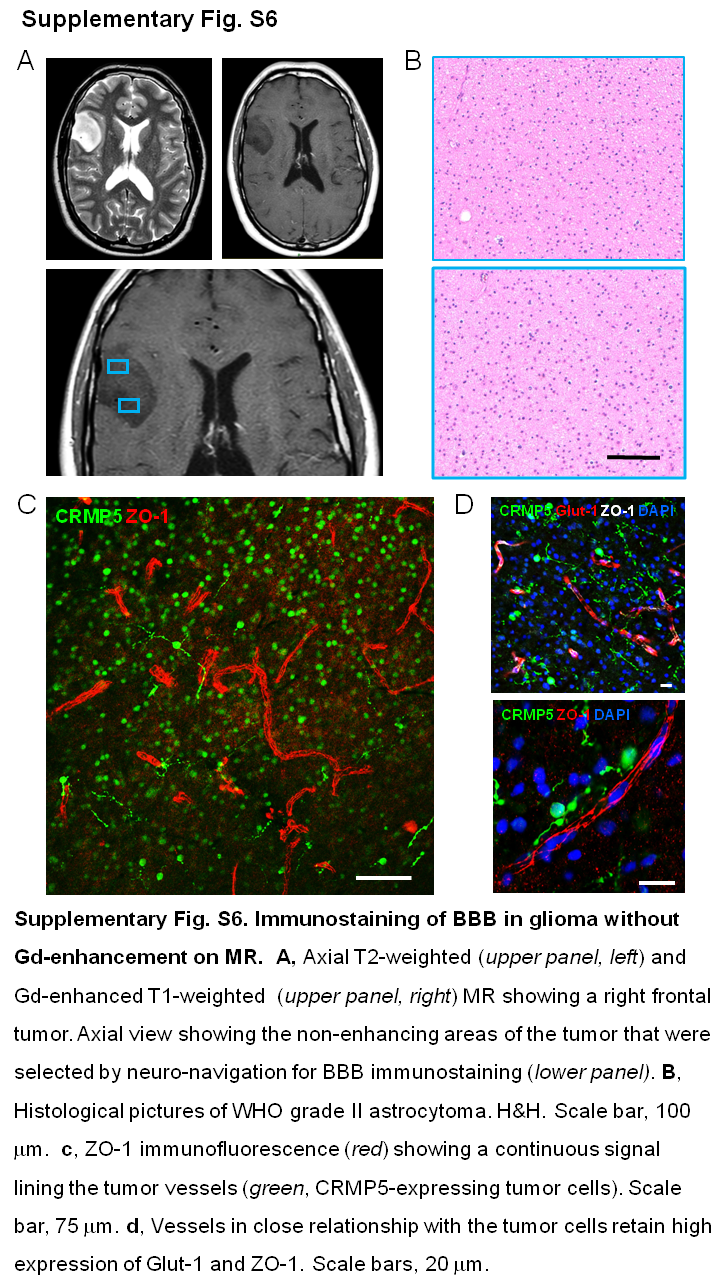

Supplement: Supplementary file 1 [file cancers-12-00018-s001.zip › Supplementary Figure S6.tif]

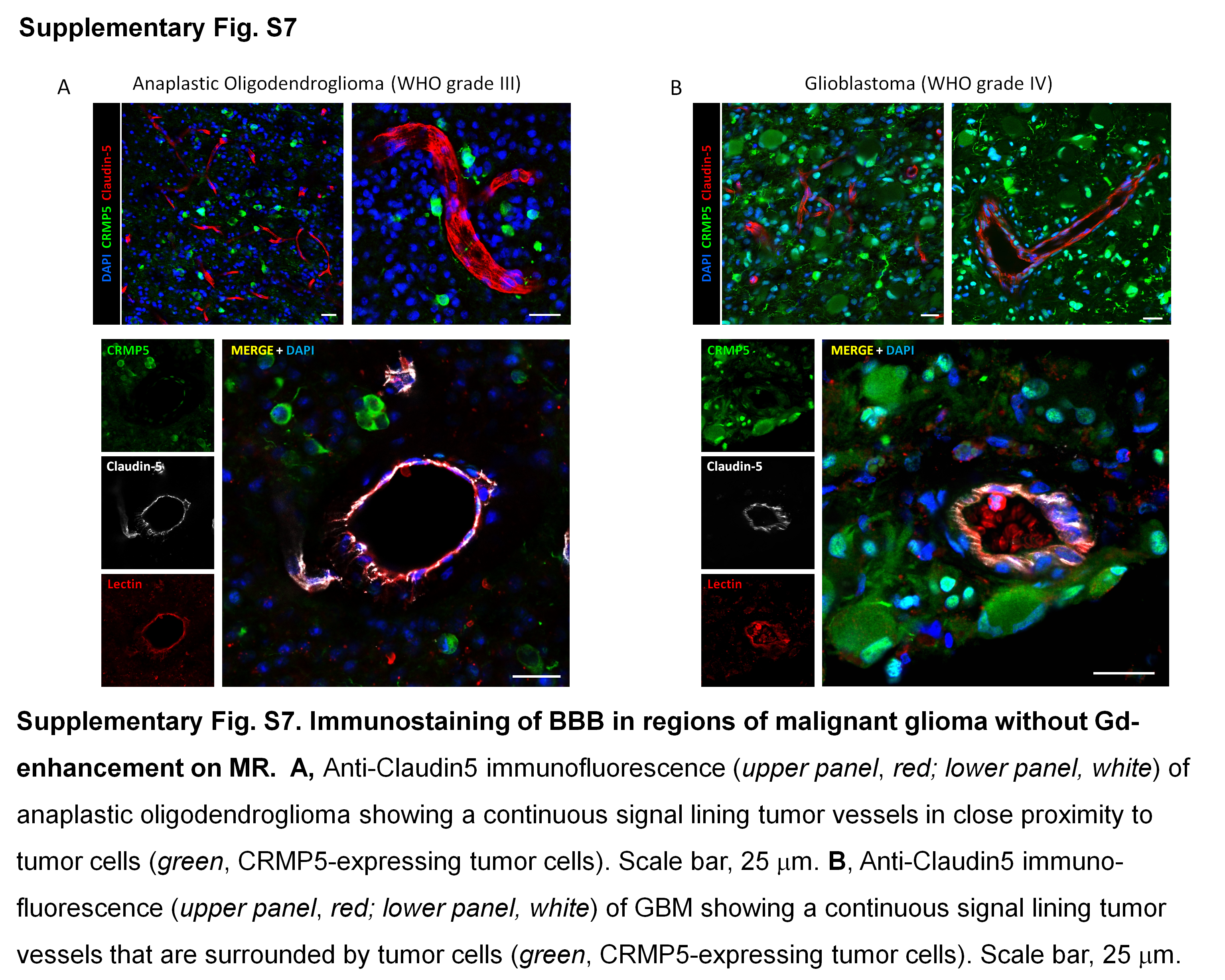

Supplement: Supplementary file 1 [file cancers-12-00018-s001.zip › Supplementary Figure S7.tif]

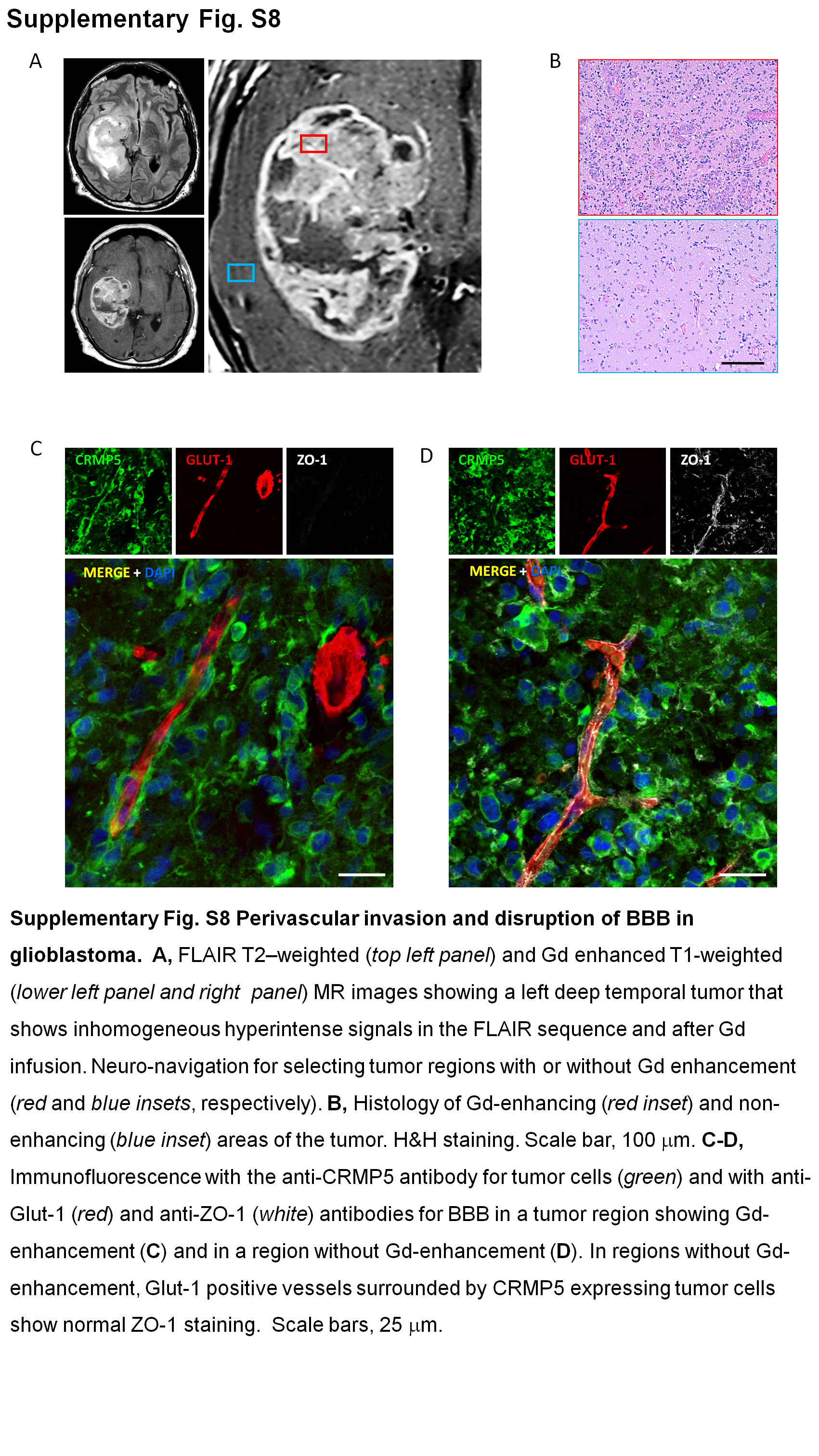

Supplement: Supplementary file 1 [file cancers-12-00018-s001.zip › Supplementary Figure S8.tif]

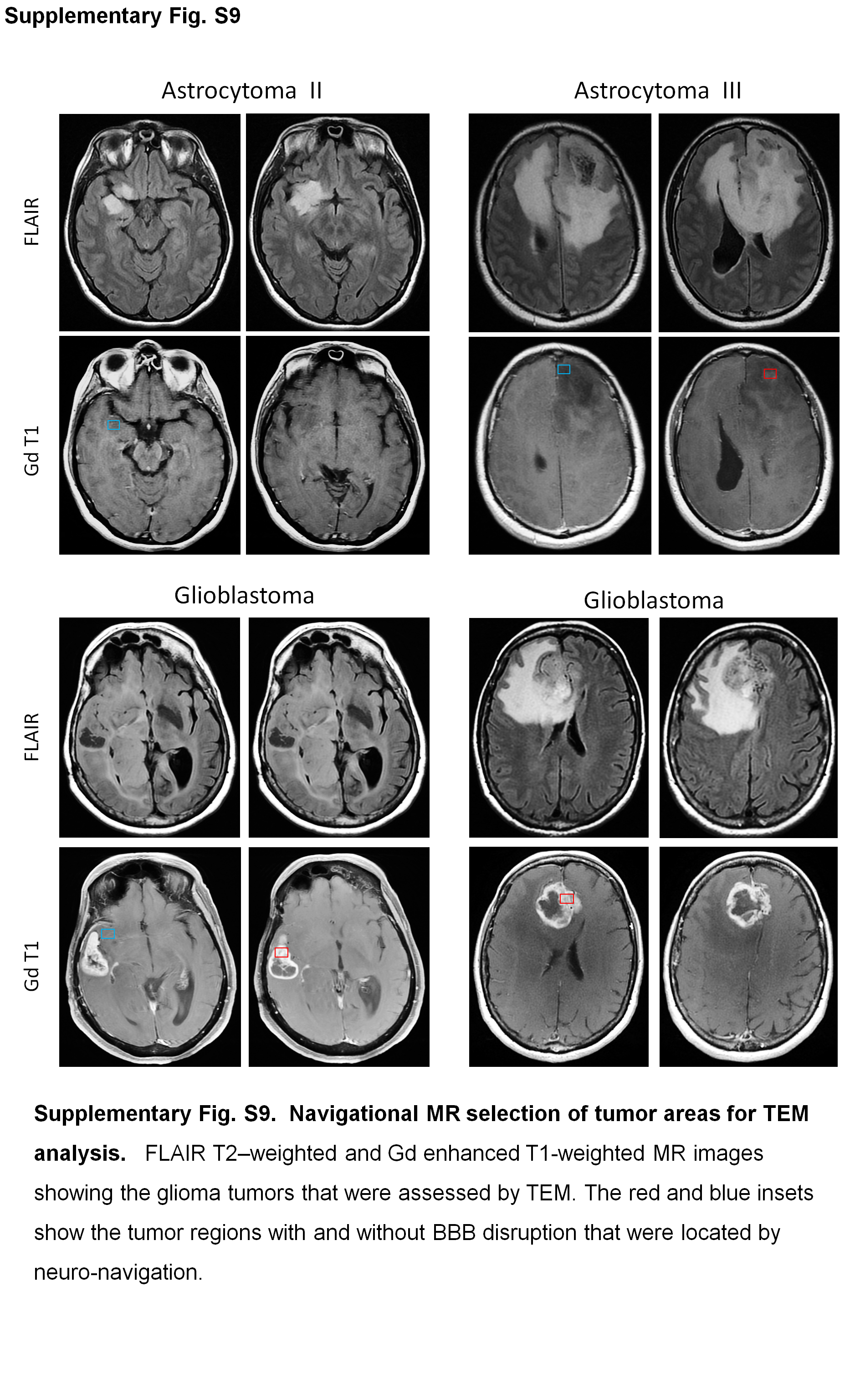

Supplement: Supplementary file 1 [file cancers-12-00018-s001.zip › Supplementary Figure S9.tif]

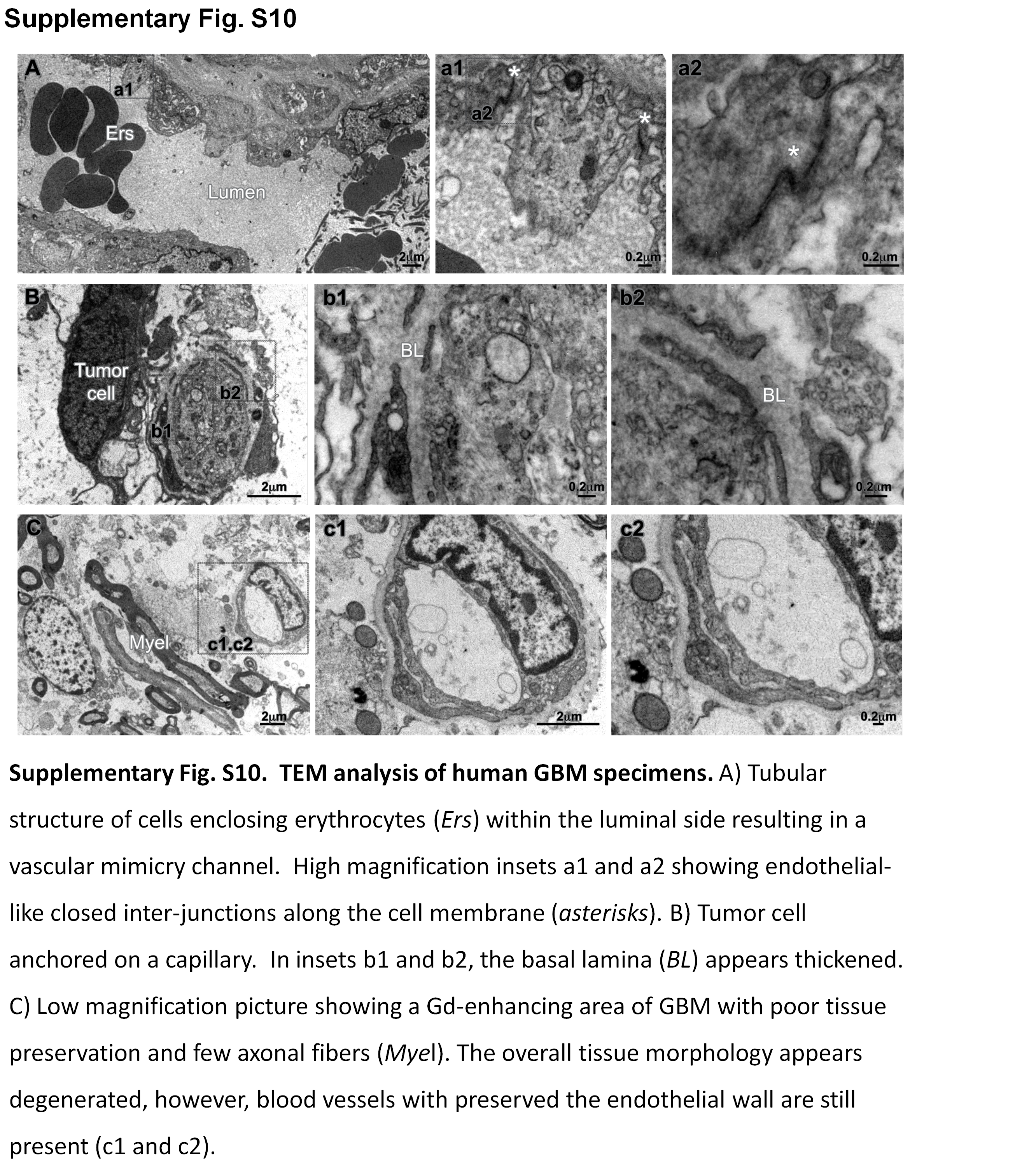

Supplement: Supplementary file 1 [file cancers-12-00018-s001.zip › Supplementary Figure S10.tif]
